# Helminth-related Eosinophilia in African Immigrants, Gran Canaria

**DOI:** 10.3201/eid1210.060102

**Published:** 2006-10

**Authors:** Javier Pardo, Cristina Carranza, Antonio Muro, Alfonso Angel-Moreno, Antonio-Manuel Martín, Teresa Martín, Michele Hernández-Cabrera, José-Luis Pérez-Arellano

**Affiliations:** * Universidad de Salamanca, Salamanca, Spain;; † Hospital Universitario de Salamanca, Salamanca, Spain;; ‡ Universidad de Las Palmas de Gran Canaria, Las Palmas de Gran Canaria, Spain;; § Hospital Universitario Insular de Gran Canaria, Las Palmas de Gran Canaria, Spain;; ¶ Hospital Universitario Insular de Gran Canaria, Las Palmas de Gran Canaria, Spain

**Keywords:** immigrants, eosinophilia, Africa, helminths

## Abstract

Of 788 recent African adult immigrants to Las Palmas de Gran Canaria, 213 (27.0%) had eosinophilia. The most frequent causes were filariasis (29.4%), schistosomiasis (17.2%), and hookworm infection (16.8%). Stool microscopy and filarial and schistosomal serologic tests gave the highest diagnostic yield. Country of origin and eosinophil count were associated with specific diagnoses.

We prospectively evaluated the prevalence and causes of eosinophilia in recent adult immigrants from Africa; the diagnostic usefulness of parasitologic and serologic tests; and the relationship between specific helminthic infections, country of origin, and degree of eosinophilia. After they gave written consent, 788 African immigrants were screened by examination of detailed medical records, physical examination, routine laboratory tests, serologic tests, the Mantoux test, and chest radiographs. Of the immigrants, 213 met the following inclusion criteria: 1) arrival within 6 months; 2) age >18 years, and 3) eosinophilia (>0.45×10^9^ eosinophils/L). Direct parasitologic tests included the examination of 3 stool samples (both Kato-Katz and Ritchie techniques were used for each sample) and specific tests for *Strongyloides stercoralis* (Baermann test and agar culture) (*1*), optic microscopy of a terminal urine specimen, and Knotts test for microfilaremia. The immune chromatographic test for *Wuchereria bancrofti* (ICT Filariasis Binax, Portland, ME, USA), skin snips, and the Mazotti test were also used in selected cases.

ELISAs with crude extracts of adult *Dirofilaria immitis* adult worm antigens (AWA Di) (*2*), *Schistosoma bovis* worm antigens (*3*), *Fasciola hepatica* excretory/secretory antigens (*4*), and *Trichinella spiralis* L1 antigens (*5*) were used. Polystyrene microtiter plates were coated with 100 μL antigens per well in carbonate buffer (pH 9.6). Serum diluted 1:100 was added and incubated for 1 h at 37°C. Horseradish peroxidase goat anti-human immunoglobulin G (Sigma, Saint Louis, MO, USA) was added at different dilutions. Washes were performed 3 times with 200 μL phosphate-buffered saline–Tween 20 per well. After incubation for 1 h at 37°C, the substrate solution (ortho-phenylenediamine-H_2_O_2_) was added, and the reaction was stopped with 3N H_2_SO_4_.

Assay sensitivities were evaluated by using serum specimens from patients with a definite diagnosis of isolated helminthic disease (Table 1). In all patients, adequate parasitologic tests showed no other helminthic infection. To evaluate specificities, we used serum samples from Spanish blood donors; samples from healthy controls from sub-Saharan Africa; and samples from patients with isolated helminthic, protozoal, bacterial, or viral infections (Table 1). Healthy controls from sub-Saharan Africa were clinically evaluated; they did not have eosinophilia, and results of a systematic investigation for helminthic infections (using stool samples, urine samples, and Knotts test) were negative.

**Table 1:** Characteristics of immunodiagnostic tests*

Test	Antigen	mg per well of antigens	Serum dilution	Anti-IgG peroxidase dilution	Sensitivity, %†	Specificity, %‡
*Schistosoma* spp.	AWA *S. bovis*	0.05	1:100	1:2,000	94	97
Filaria	AWA *Dirofilaria immitis*	0.08	1:100	1:5,000	90	97
*Fasciola* spp.	E/S *F. hepatica*	0.04	1:100	1:2,000	100	96
*Trichinella* spp.	L1 *T. spiralis*	0.03	1:100	1:2,500	100	91

*IgG, immunoglobulin G; AWA, adult worm antigens; E/S, excretory/secretory antigens; L1, larvae 1 antigens. †Sensitivity: serum samples from patients infected with schistosomiasis (35), tropical filariasis (20), fascioliasis (12), and trichinellosis (3) were used. ‡Specificity: serum samples from healthy controls from sub-Saharan Africa (41), from healthy Spanish blood donors (52), from patients with other isolated helminthic infections (45), from patients with protozoa infections (25), and from patients with bacterial or viral infections (19) were used.

Moreover, an ELISA was used to test for strongyloidiasis with somatic larvae antigens from *Strongyloides venezuelensis*. Although the ELISA is 100% sensitive, its low specificity precluded its use as a diagnostic tool.

The SPSS 11.5 statistical package (available from http://www.spss.com) was used for analyses. The level of significance accepted was <0.05, and results were expressed as means plus standard deviation (SD). The receiver-operating-characteristic curve was used to establish ELISA cut-offs. The χ^2^ and the Fisher exact tests were used to evaluate the association between demographic variables and final diagnoses, and the Student *t* test was used to compare the degree of eosinophilia among patients with single and multiple infections. Analysis of variance and post-hoc tests were used to compare the mean eosinophil counts in each final diagnosis.

We found that 213 (27.0%) of 788 immigrants whose conditions were analyzed had eosinophilia. Of these, 191 (89.7%) were male, with a mean age of 27.4 years (SD 8.3). Two hundred two (94.9%) patients were from sub-Saharan countries, mainly Nigeria (24.1%), Sierra Leone (17.3%), Ghana (15.0%), and Mali (8.9%); 165 (77.1%) patients had 0.450–0.999×10^9^ eosinophils/μL, 47 (21.9%) had 1.000–2.999×10^9^ eosinophils/μL, and 1 patient had >3.000×10^9^ eosinophils/μL.

One hundred fifty-four study participants (72.3%) were asymptomatic. In symptomatic patients (28.0%), the most frequent clinical features were lymphadenopathy (6.1%), pruritus (5.6%), and skin lesions (3.3%).

A final diagnosis was made in 161 cases (75.6%): 116 (54.5%) had 1 parasite, 30 (14.1%) had 2, and 15 (7.0%) had >3. The most frequent parasites were filariae (n = 63, 29.6%), schistosomes (n = 37, 17.4%), hookworms (n = 36, 16.8%), and *Trichuris* spp. (n = 18, 8.4%) (Figure 1). Direct methods were used in 60 (37.2%) patients, indirect methods were used in 80 (49.6%), and both methods in 21 (13.0%) patients. Stool microscopy and filarial and schistosomal serologic testing yielded the highest positive result rates (Table 2). The country of origin was statistically associated (p<0.05) with the final diagnosis: 77% of the patients with eosinophilia from Cameroon had filariasis, 63% of the patients from Mali had schistosomiasis, and 30.8% of the patients from Nigeria had hookworm infection.

**Figure 1:**
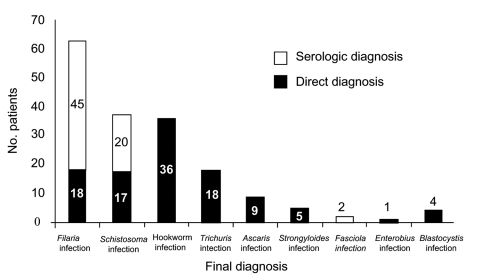
Final diagnosis of patients with eosinophilia. Filarial species detected by direct methods were *Mansonella perstans* (n = 13), *Loa loa* (n = 4), and *Onchocerca volvulus* (n = 1). Schistosomal species diagnosed by direct methods were *Schistosoma hematobium* (n = 10), *S. mansoni* (n = 6), and *S. intercalatum* (n = 1).

**Table 2:** Diagnostic yield of etiologic tests*

Test	Test done, no. (*%*)	Yield of test, %
Stool (microscopy)	175 (81)	35
Filarial serology	189 (92)	30
*Schistosoma* spp. serology	213 (100)	28
Urine (microscopy)	66 (30)	16
Knotts test	123 (57)	13
*Trichinella* spp. serology	208 (97)	11
*Fasciola* spp. serology	209 (97)	7
ICT *Wuchereria bancrofti*	71 (33)	4

*ICT, immune chromatographic test.

The mean eosinophil count was significantly higher in patients with a final diagnosis than in those whose conditions were not diagnosed (871 ± 431 vs. 643 ± 179) (p<0.05), and the mean count was higher also in patients with 2 or more parasites than in patients with 1 (1,045 ± 641 vs. 827 ± 389) (p<0.05). Among patients with 1 helminthic disease, those with filariasis had higher eosinophil counts than those with schistosomiasis or geohelminthic infection (p<0.05) (Figure 2).

**Figure 2:**
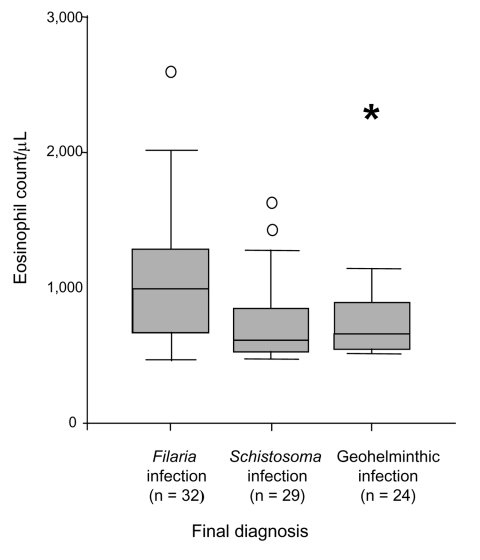
Relationship between eosinophil counts and the parasitologic diagnosis. Data are expressed as a box-and-whisker plot showing median, interquartile range (IQ), and extreme values. Circles indicate atypical outliers (values 1.5–3×IQ), and asterisk represents extreme outliers (values >3×IQ).

Eosinophilia is frequent in travelers and expatriates from tropical areas (*6**–**12*). However, its prevalence is variable (3.1%–50%), depending on the population studied (more frequent in immigrants than in travelers), the areas where infection occurs (mainly sub-Saharan Africa or Southeast Asia), and the design of the study (prospective or retrospective). In this prospective work, we studied a homogeneous population of immigrants who had recently arrived from Africa, and we detected eosinophilia in 27%.

Studies of persons with imported eosinophilia have made a diagnosis that identified the etiologic agent in 15% to 64% of cases (depending on the population, the selected eosinophil count, and the methods) (*6**–**13*). Using direct and serologic methods (*10**,**13*), we detected helminthic infections in 75% of the patients. In all series, the main diagnoses are filarial, schistosomal, and geohelminthic infections. Only 27.7% of our patients had related signs or symptoms, which indicates that a proper investigation can detect many asymptomatic infections.

The sensitivities of our serologic tests were >90%, with specificities of 85%–97%. Using *D. immitis* antigens for the immunodiagnosis of tropical filariasis (*14*), we obtained a sensitivity of 90% for microfilaremia, with 97% specificity. The utility of adult worm antigens of *S. bovis* for serodiagnosis of schistosomiasis has been recently demonstrated (*3*).

Our high diagnostic yield with filarial (30%) and schistosomal (28%) serologic testing is similar to that obtained by Whetham et al. in travelers returning from West Africa (*10*). Among the direct methods, stool microscopy was the most sensitive (35%). However, serologic testing detected another parasitic infection (mainly filarial or schistosomal) when direct tests showed only a geohelminthic infection (13.2%), which suggests that direct and indirect tests are complementary in this population.

The proportion of *Strongyloides* spp. infection diagnosed was lower than in almost all other similar studies (*6**–**12*) because we could not ascertain it by stool positivity only, because of the low specificity of *Strongyloides* serologic testing available to us. Patients from Mali with eosinophilia had schistosomiasis more frequently, as reported in some European studies (*15*). However, we found a significant correlation between filarial or hookworm infection and immigration from Cameroon and Nigeria, respectively, an association not described previously. Finally, filariasis induces higher eosinophil counts than other parasitic infections, likely because the parasite inhabits blood and tissue and is not limited to the gut lumen. Our results show that 1) eosinophilia is frequent in recently arrived African immigrants, 2) helminthic infections can be diagnosed by using both parasitologic and serologic tests, 3) an immigrant's country of origin may suggest specific parasitic diseases, and 4) higher eosinophil counts usually indicate filariasis.

## References

[1] Siddiqui AA, Berk SL. Diagnosis of *Strongyloides stercoralis* infection. Clin Infect Dis. 2001;33:1040–7. 10.1086/322707 11528578

[2] Perera L, Pérez Arellano JL, Cordero M, Simón F, Muro A. Utility of antibodies against a 22 kDa molecule of *Dirofilaria immitis* in the diagnosis of human pulmonary dirofilariosis. Trop Med Int Health. 1998;3:151–5. 10.1046/j.1365-3156.1998.00209.x 9537278

[3] Pardo J, Carranza C, Turrientes MC, Pérez Arellano JL, Lopez-Velez R, Ramajo V. Utility of *Schistosoma bovis* adult worm antigens for diagnosis of human schistosomiasis by enzyme-linked immunosorbent assay and electroimmuno-transfer blot techniques. Clin Diagn Lab Immunol. 2004;11:1165–70. 15539523 10.1128/CDLI.11.6.1165-1170.2004 PMC524774

[4] Hillyer GV, Soler de Galanes M. Identification of a 17-kDa *Fasciola hepatica* immunodiagnostic antigen by enzyme-linked immunoelectrotransfer blot technique. J Clin Microbiol. 1988;26:2048–53. 3182993 10.1128/jcm.26.10.2048-2053.1988 PMC266814

[5] Alcántara P, Correa D. Human humoral immune response against *Trichinella spiralis.* Int J Parasitol. 1993;23:657–60. 10.1016/0020-7519(93)90173-V 8225769

[6] Harries AD, Myers B, Bhattacharrya D. Eosinophilia in Caucasians returning from the tropics. Trans R Soc Trop Med Hyg. 1986;80:327–8. 10.1016/0035-9203(86)90049-0 3787694

[7] Nutman TB, Ottesen EA, Ieng S, Samuels J, Kimball E, Lutkoski M, Eosinophilia in Southeast Asian refugees: evaluation at a referral center. J Infect Dis. 1987;155:309–13. 10.1093/infdis/155.2.309 3805765

[8] Libman MD, MacLean JD, Gyorkos TW. Screening for schistosomiasis, filariasis, and strongyloidiasis among expatriates returning from the tropics. Clin Infect Dis. 1993;17:353–9. 10.1093/clinids/17.3.353 8218675

[9] Schulte C, Krebs B, Jelinek T, Nothdurft HD, von Sonnenburg F, Loscher T. Diagnostic significance of blood eosinophilia in returning travelers. Clin Infect Dis. 2002;34:407–11. 10.1086/338026 11753824

[10] Whetham J, Day JN, Armstrong M, Chiodini PL, Whitty CJ. Investigation of tropical eosinophilia; assessing a strategy based on geographical area. J Infect. 2003;46:180–5. 10.1053/jinf.2002.1108 12643868

[11] Lopez-Velez R, Huerga H, Turrientes MC. Infectious diseases in immigrants from the perspective of a tropical medicine referral unit. Am J Trop Med Hyg. 2003;69:115–21. 12932108

[12] Seybolt LM, Christiansen D, Barnett ED. Diagnostic evaluation of newly arrived asymptomatic refugees with eosinophilia. Clin Infect Dis. 2006;42:363–7. 10.1086/499238 16392081

[13] Whitty CJ, Carroll B, Armstrong M, Dow C, Snashall D, Marshall T, Utility of history, examination and laboratory tests in screening those returning to Europe from the tropics for parasitic infection. Trop Med Int Health. 2000;5:818–23. 10.1046/j.1365-3156.2000.00642.x 11123831

[14] Dumenigo Ripoll B, Espino Hernandez AM, Menendez Valonga MC, Finlay Villalvilla C. Excretion-secretion antigens from adult *Dirofilaria immitis* in the diagnosis of human filariasis by solid phase immunoenzyme assay. Rev Cubana Med Trop. 1991;43:162–6. 9768181

[15] Grobusch MP, Muhlberger N, Jelinek T, Bisoffi Z, Corachan M, Harms G, Imported schistosomiasis in Europe: sentinel surveillance data from TropNetEurop. J Travel Med. 2003;10:164–9. 10.2310/7060.2003.35759 12757691

